# 

**Published:** 1862-12

**Authors:** 


### CONTENTS.

### ORIGINAL CONTRIBUTIONS.

ART. XXXIX. SILVER MIXTURE AND NERVOUS SEDATIVES. By Ira Hatch, M.D., ........ 715

ART. XL. PLACENTA PRÆVIA. By Dr. R. B. Treat, of Janesville, Wis., ........ 721

ART. XLI. A FATAL CASE OF URÆMIA. Reported by Addison Niles, M.D., Quincy, Ill., ........ 724

ART. XLII. TWO CASES OF FRACTURE OF THE SKULL. By Hiram Wanzer, M.D., Chicago, Ill., ........ 726

ART. XLIII. PRACTICE OF MEDICINE AND SURGERY IN MODERN TIMES; SINS AND DELINQUENCIES OF PROFESSIONAL MEN, ........ 728

### SELECTIONS.

Ovariotomy, ........ 732

Experimental Researches into a new Excretory Function of the Liver; Consisting in the Removal of Cholesterine from the Blood, and its Discharge from the Body in the form of Stercorine. (*The Seroline of Boudet.*) By Austin Flint, Jr., M.D., Professor of Physiology and Microscopy in the Bellevue Medical College, New York; &c... 733

### EDITORIAL.

Letter from Dr. McFarland, Jacksonville, Ill., ........ 763

Death of Dr. George Coatsworth, ........ 772

Explanations, ........ 773

The Future, ........ 773

Change in a well-known Drug Store, ........ 773

Circular, No. 13, ........ 773

